# Study protocol of the YOU CALL - WE CALL TRIAL: impact of a multimodal support intervention after a "mild" stroke

**DOI:** 10.1186/1471-2377-10-3

**Published:** 2010-01-06

**Authors:** Annie Rochette, Nicol Korner-Bitensky, Duane Bishop, Robert Teasell, Carole White, Gina Bravo, Robert Côté, Jean Lachaine, Teri Green, Louise-Hélène Lebrun, Sylvain Lanthier, Moira Kapral, Sharon Wood-Dauphinee

**Affiliations:** 1 Montreal University, Montreal, Quebec, Canada; 2 Centre for Interdisciplinary Research in Rehabilitation of Greater Montreal, Quebec, Canada; 3 McGill University, Montreal, Quebec Canada; 4 St-Lukes Hospital, Rhode Island, USA; 5 University of Western Ontario, Ontario, Canada; 6 University of Texas, San Antonio, USA; 7 Sherbrooke University, Quebec, Canada; 8 Research Center on Aging, Sherbrooke, Quebec, Canada; 9 University of Calgary, Alberta, Canada; 10 University of Toronto, Ontario, Canada

## Abstract

**Background:**

More than 60% of new strokes each year are "mild" in severity and this proportion is expected to rise in the years to come. Within our current health care system those with "mild" stroke are typically discharged home within days, without further referral to health or rehabilitation services other than advice to see their family physician. Those with mild stroke often have limited access to support from health professionals with stroke-specific knowledge who would typically provide critical information on topics such as secondary stroke prevention, community reintegration, medication counselling and problem solving with regard to specific concerns that arise. Isolation and lack of knowledge may lead to a worsening of health problems including stroke recurrence and unnecessary and costly health care utilization.

The purpose of this study is to assess the effectiveness, for individuals who experience a first "mild" stroke, of a sustainable, low cost, multimodal support intervention (comprising information, education and telephone support) - "***WE CALL***" compared to a passive intervention (providing the name and phone number of a resource person available if they feel the need to) - "***YOU CALL***", on two primary outcomes: unplanned-use of health services for negative events and quality of life.

**Method/Design:**

We will recruit 384 adults who meet inclusion criteria for a first mild stroke across six Canadian sites. Baseline measures will be taken within the first month after stroke onset. Participants will be stratified according to comorbidity level and randomised to one of two groups: YOU CALL or WE CALL. Both interventions will be offered over a six months period. Primary outcomes include unplanned use of heath services for negative event (frequency calendar) and quality of life (EQ-5D and Quality of Life Index). Secondary outcomes include participation level (LIFE-H), depression (Beck Depression Inventory II) and use of health services for health promotion or prevention (frequency calendar). Blind assessors will gather data at mid-intervention, end of intervention and one year follow up.

**Discussion:**

If effective, this multimodal intervention could be delivered in both urban and rural environments. For example, existing infrastructure such as regional stroke centers and existing secondary stroke prevention clinics, make this intervention, if effective, deliverable and sustainable.

**Trial Registration:**

ISRCTN95662526

## Background

### What is a mild stroke?

An extensive search of the medical literature from 1966 to 2008 through Medline, CINAHL, Current Content, Evidence Based Medicine (EBM) Reviews was conducted on the topic of "mild" stroke. Although there is no clear definition of "mild" stroke, there is *general consensus *that a stroke is classified as "mild" when motor function or ability to accomplish personal activities of daily living is only minimally affected (modified Rankin score 0-to-2) [1]. Using the Canadian Neurological Scale, between April 1995 and March 1997, Jones and collaborators categorized as mild (scores 8.5 to 11.5) more than 60% of 984 male veterans from a prospective cohort of individuals with stroke admitted acutely to any of nine geographically diverse Veterans Administration Hospitals within the United States [2]. More recently, the prospective NEMESIS cohort study conducted in Australia [3] reported 67% of 219 two-year stroke survivors had an initial National Institutes of Health Stroke Scale (NIHSS) score between 0-to-5 indicating mild initial stroke severity. Despite the high prevalence of mild stroke, very few studies have specifically addressed this subgroup, who until recently were considered to be "without sequelae".

### Stroke recurrence and secondary prevention

Stroke recurrence is a major issue. The fear of another stroke is overwhelming [4] particularly shortly after stroke onset. As part of the coping process, individuals typically look for a cause of their stroke (causal attributions) in order to decrease the risk of a recurrence. They may look for internal (hypertension, lifestyle factors) and/or external causes (certain events, fate). The results of a recent study examining causal attributions and health behaviour changes suggest that individuals who express external attributions demonstrate poorer health behaviour choices than those who express internal attributions [5]. Therefore, adherence to the required changes in lifestyle, which is necessarily demanding, will be influenced by the person's perception of what caused his or her stroke [6]. Timely information and support to those with mild stroke and who fear a recurrence is thus needed to facilitate a change in lifestyle.

According to the Secondary Stroke Prevention module of the Stroke Rehabilitation Evidence-Based Review (SREBR) [7] led by one member of this research team (RT), adequate monitoring and management of risk factors post-stroke is essential to minimise the risk of subsequent stroke. In addition to strong evidence (level 1A) of the effectiveness of adequate management and monitoring of specific conditions such as high blood pressure or diabetes [8,9]., promotion of "healthy behaviours" and lifestyle changes will have a positive effect on stroke prevention. There is moderate evidence that behavioural intervention can reduce the risk of stroke [7]. Although lifestyle changes are difficult to adopt, Redfern and collaborators report extremely positive results of a prospective study conducted from 1995 and 1998 that found, despite evident problems in continuity and effectiveness of care offered to 717 individuals with a first-time stroke, that by one-year post stroke, 41.4% of smokers had quit, 85% of excessive alcohol drinkers had reduced their consumption to below recommended weekly limits, and 41.1% of obese patients were no longer obese. Most of these changes occurred in the first three months post-stroke [10]. Generally, actual knowledge of risk factors, symptoms, and treatment, remains poor even for individuals who have already sustained a stroke [11] supporting the necessity of addressing this unmet need through an intervention aimed at the promotion of secondary prevention.

### Consequences of "mild" stroke

In 1997, Duncan and coworkers [12] compared health status of individuals with a mild stroke (n = 304) to two groups: 184 people with transient ischemic attack (TIA) and 654 people without a history of stroke/TIA but at elevated risk for stroke-asymptomatic group. The findings indicated that the consequences of a mild stroke affected all dimensions of health except pain, and that those with stroke were significantly more impaired in physical functioning, and in physical roles such as work or leisure, when compared to the other two groups. A year later, in a randomized controlled pilot study, Duncan and co-workers reported positive effects on motor function of a home-based exercise program for individuals with mild and moderate stroke [13]. Another study from Sweden indicated that life satisfaction was significantly below norms especially satisfaction with life as a whole, sex life, and ability to manage self-care, at one-year follow-up in individuals less than 75 years of age with "mild" stroke (average Barthel Index score of 99.5/100 ± 0.5) [1]. More recently, two qualitative studies have reported "hidden" [14] or "invisible" [15] consequences of stroke such as fatigue being an important source of frustration that impacts negatively on work, family, and social life. Another study led by two members of our team (AR, GB), using both quantitative and qualitative methodologies, has shown a marked decrease in participation in daily activities and social roles in individuals who sustained a "mild" stroke (n = 35) [16]. Statistically and clinically significant restrictions in participation persisted even six months post-stroke compared to pre-stroke levels particularly related to driving, community life, leisure activities, employment, and relationships. Furthermore, more refined analyses of this sample have demonstrated that these individuals present higher risks of depressive symptoms then that of the general stroke population [17]: in the first two weeks post-stroke, 28.6% obtained a score ≥ 10 on the Beck Depression Inventory [18,19],. 32.4% at three months post-stroke and 20.0% at six months post-stroke, suggesting the presence of depressive symptoms. In 2002, Martin and colleagues assessed six month outcomes including unmet needs and adherence to secondary prevention advice in a follow up study of 208 individuals with an acute stroke (87.5% of participants experienced mild stroke) [20]. Issues raised by participants included a feeling of being abandoned by the healthcare system following hospital discharge, poor access to psychological support, lack of confidence in resuming social activities (even in those with a good physical recovery), altered role changes within the family and an intense fear of another stroke. In a review of this topic, Rodgers and coworkers [21] concluded that affected individuals and their families had a desire for further knowledge about the causes and consequences of stroke, secondary preventative measures, and the availability of support (formal and informal) in the community.

In summary, there is the misconception that a mild stroke results in no or minimal sequelae with mounting evidence that "mild" stroke causes important consequences on all levels of health and results in the same high risk of negative events as severe stroke. This subgroup of individuals, being the most prevalent of all stroke groups, requires greater research to determine their unique needs and how those needs can be best addressed.

### Potential benefits of provision of information and support

According to the Stroke Rehabilitation Evidence-Based Review (SREBR) [22], "*there is strong (Level 1a) evidence of a positive benefit associated with the provision of information and education through a variety of intervention types*" (p.36). Indeed, three of the six RCTs were considered of high quality [23-25]. (Pedro score ≥ 6), the other three being of fair quality [26-28] These interventions include the **provision of an information package **with a positive effect on stroke knowledge [23,27]. and on quality of life [23]. **Education sessions **have been shown to be effective on improving stroke knowledge [24,28] and enhancing satisfaction with information [25] as well as decreasing depression and increasing self-efficacy [26,28] More recently, a pilot study of an intervention developed by one member of the team (DB) that includes **telephone support **(***FITT: Family Intervention Telephone Tracking***) when compared to usual care, showed a significant decrease in health care services utilisation with an increase in quality of life for individuals who sustained a stroke (Bishop DS, Miller I, Weiner D, Guilmette T, Mukand J, Evans RL, et al. Family Intervention: Telephone Tracking (FITT): A preliminary stroke outcome study. Submitted). Furthermore, telephone care was shown to be significantly less costly and more accessible than clinic-based care particularly for people who have limited access to transportation or who live in a rural region [29].

### What about the use of technology to provide information?

Between 2000 and 2003, there was an increase of more than 20% in home internet users in the US population aged 65 and over and use is expected to keep increasing [30]. With advances in technology, patients are increasingly turning to the Internet for medical information [31]. Their main reasons for performing Internet searches are to seek information about health conditions, symptoms, and treatments [32]. To answer the needs of patients and their families on stroke rehabilitation information, ***StrokEngine ***http://www.medicine.mcgill.ca/strokengine was developed in English and French. *StrokEngine *is a point-of-care website that includes the 'A to Z' (Acupuncture, Bobath, Constraint-induced therapy, Driver retraining, etc.) of rehabilitation interventions for stroke in an easy to use, easy to understand web-based format. Every intervention used by rehabilitation professionals for treating individuals with stroke in Canada is accessible from a one-page StrokEngine Desk Top Display. Printer-friendly handouts are available for clients and families. The development of *StrokEngine *is led by two members of the team (NKB and RT). It includes, for every intervention, a summary of evidence, an in-depth review and a "*family/patient information section*" describing the main findings in an easy to understand layman's terminology. The assessment of the usability and navigability of the Family/Patient component of StrokEngine has shown that in addition to being easy to use it is highly welcomed by clients and their families [33]. The provision of telephone support should help individuals under stress navigate quickly to the appropriate section of the website.

### ICF as a conceptual model

This research proposal uses the International Classification of functioning, disability and health (ICF) as its framing construct. A "mild" stroke represents the *health condition *creating a change in *body function and structures *including cognitive, visual, perceptual or physical impairments, limitations in *activities *and restrictions in life involvement (*participation*) such as driving, work and recreation activities. It is hypothesised that without timely information and support to adequately reduce risk factors and adapt to this new health condition, negative outcomes such as unplanned visits to the health care system for negative events and decreased quality of life, will occur.

Therefore, the proposed multimodal support intervention would act as a ***facilitator ***being part of the *service, system and policy *component of *environmental factors*. It should minimize the negative consequences of a "mild" stroke through the provision of individualised information to the person (*personal factors*) about their *health condition *and on how to concretely minimise the risk of another stroke in the future (secondary prevention), as well as through the provision of support and assistance to optimally cope and adapt with their new condition. Consequently, the proposed intervention should have an effect on the following outcomes: decrease unplanned-use of the health care system (*environmental factors*) for negative events through adequate monitoring of risk factors (*health condition*) and concomitantly, improve quality of life by reducing the risk of depression and recurrent stroke (*body functions and structures*) and by enhancing *participation*.

### The research objective

The purpose of this study is to assess the effectiveness, for individuals who experience a first "mild" stroke, of a sustainable, low cost, multimodal support intervention (comprising information, education and telephone support) - "***WE CALL***" compared to a passive intervention (providing the name and phone number of a resource person available if they feel the need to) - "***YOU CALL***", on two primary outcomes: unplanned-use of health services for negative events and quality of life. Secondary outcomes include participation level, depressive symptoms, and planned-use of health services for health promotion and secondary prevention. A secondary objective is to *explore *the contribution of potential explanatory variables such as age, gender, living alone versus with a significant other, comorbidity level and access (or not) to a secondary stroke prevention clinic, on outcomes. We hypothesize that the provision of the *"WE CALL" *intervention over the first six months post-stroke, compared to the *"YOU CALL" *will have three positive effects persisting 6 months beyond the intervention period:

1) A decreased unplanned-used of health services for negative events;

2) An improved quality of life;

3) Less depressive symptoms, a better participation in daily activities and social roles, and an increase in planned health visits for promotion and secondary stroke prevention;

In addition, we hypothesize that:

4) Participants with higher comorbidity level will benefit more from the active intervention given the increased complexity of their health status and,

5) Participants with access to secondary stroke prevention (SPC) clinic will benefit more from the intervention than those without SPC access.

In summary, in most cases, individuals with a "mild" stroke are discharged home directly from the acute care hospital without any further referrals to rehabilitation professionals. A number of qualitative studies have shown that, for individuals with stroke, information and support is needed [34,35] with timing and type of information dependent on the individual's current life situation. Indeed, the World Health Organisation advises "*patients have a right to be given factual, supportable, understandable and appropriate information*". Yet, within the current Canadian health care system, individuals with "mild" stroke have limited access to adequate information and support, even in the face of mounting evidence of their effectiveness on mental health [26], stroke knowledge [25] and quality of life (Bishop DS, Miller I, Weiner D, Guilmette T, Mukand J, Evans RL, et al. Family Intervention: Telephone Tracking (FITT): A preliminary stroke outcome study. Submitted). Therefore, this trial aims to determine if a sustainable, low cost multimodal support intervention ("*we call*") is effective compared to the availability of a resource person ("*you call*") in reducing unplanned use of the health care system for negative events and on improving quality of life for individuals who experience a first "mild" stroke.

## Methods/Design

### Design

**Figure 1:**
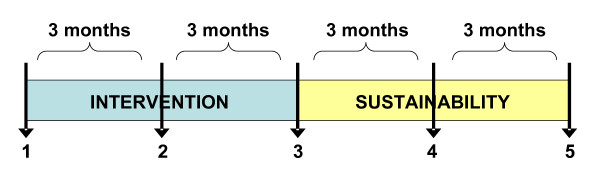
**Study plan**. 1. Informed consent, baseline assessment (primary and secondary outcomes) and randomisation; 2. Three-month primary outcomes assessment time; 3. Post- intervention assessment time (primary & secondary outcomes); 4. Self- reported use of health services assessment (from month #6 to #9); 5. Follow- up assessment (primary & secondary outcomes).

We are conducting a randomized clinical trial, over the first six months post-stroke, with a follow-up at one year (see figure 1). Multi-site recruitment across three Canadian provinces, Québec (Montréal), Ontario (Brantford, Chatham and Kitchener-Waterloo) and Alberta (Calgary), allows a representative sample of participants exposed to different levels of services post stroke such as differing access to Secondary Stroke Prevention Clinics. The target population for this study is all individuals/adults who sustained a first "mild" stroke and who are discharged home within three weeks of being admitted to an acute care hospital. Daily chart reviews of all new stroke admissions are being performed by a site-designated-research-nurse (who is not the treating clinician) to identify potential first mild stroke patients. Potential participants are then approached for further screening. Eligible and interested patients are requested to provide informed consent, and receive a copy of the consent form. Participants are being assessed at baseline (quality of life, participation and depression) in the first month post-stroke by way of a telephone interview. Phone interviews were shown to be as reliable as face-to-face interviews when measuring variables as complex as functional status [36]. They are then being allocated randomly, using stratified block randomization, to one of two groups: 1) "WE CALL" or 2) "YOU CALL". Stratification is done according to level of comorbidity (no or low comorbidity versus high as indicated by a score ≥ 4 on the Comorbidity Index [37]) as this variable is important to the primary outcomes: unplanned-use of health services for negative events and quality of life. Furthermore, to consider the potential effect on outcomes of availability of secondary prevention clinic and urban versus rural areas, parallel randomization (separate randomization) occurs for each province.

The interventions are being delivered from Montreal (Québec) with close collaboration with a site-designated nurse for the identification of local community resources. Participants are blind to the underlying hypotheses being tested but cannot be blinded to the intervention. Outcome assessors are blind to group assignment.

### Interventions

**WE CALL **is a multimodal (Telephone, Internet and Paper) support intervention provided to participants randomly allocated to the "**WE CALL**" group. The telephone component of the intervention is based on the Family Intervention Telephone Tracking model (FITT), where pilot data shows promising effectiveness in the US (Bishop DS, Miller I, Weiner D, Guilmette T, Mukand J, Evans RL, et al. Family Intervention: Telephone Tracking (FITT): A preliminary stroke outcome study. Submitted). The FITT focuses on reinforcing problem solving skills through counselling and favours the use of available community resources. Issues pertaining to secondary prevention and adaptation are included. Each telephone interaction focuses on any new, or ongoing issues as well as six key areas (i) family functioning, (ii) depression, (iii) neurocognitive functioning, (iv) functional independence, (v) physical health and vi) individualised risk factors.

Participants are being asked how they are doing with these six key areas. For example, when discussing functional capacity, one important issue may be driving. Did they receive any advice in regards to driving? Do they have the required abilities to drive safely? Do they need an assessment or re-training? As an intervention, they could be referred to the *StrokEngine *module specifically on driving after a stroke (via Internet or through hard copies) and specific concerns could be individually discussed over the phone. Each specific issue is addressed during the next call to ensure active involvement. Participants of this group will be called by the Trained Health Care Professional (THCP) on a weekly basis for the first two months, bi-weekly during the third month, and monthly for the last three months of the intervention. It is estimated that each intervention call should last between 15 - 20 minutes (Bishop DS, Miller I, Weiner D, Guilmette T, Mukand J, Evans RL, et al. Family Intervention: Telephone Tracking (FITT): A preliminary stroke outcome study. Submitted). Participants are encouraged to contact the THCP between intervention calls, should they feel the need to. If the THCP is unavailable, the participant is invited to leave a voice message and the THCP will contact them within the day, with the exception of weekends. The frequency and content of these additional calls is documented.

Additional written information will be provided when and as needed. Participants will be referred to local community services as necessary and/or directed to their family doctors when they experience health problems (including depression). Written information on secondary stroke prevention and effectiveness of rehabilitation interventions post-stroke will be made available either directly via an Internet website - StrokEngine - or through CD's or paper copies, for those who do not have easy access to the Internet or a computer.

**YOU CALL **group participants are provided with the name and phone number of a THCP who is not involved in providing the "we call" intervention, whom they are free to contact should they feel the need to. This THCP is different from the ones providing the intervention to the "we call" group to minimize potential contamination between the interventions due to THCPs. THCP-you call is instructed to provide only information on topics initiated by the participant. He/she is instructed to provide complete information to adequately answer participants' requests but does not probe on other issues. The use of this intervention as a control, which is very ecological (most individuals are provided with stroke information pamphlets and phone numbers to contact), is more acceptable ethically than a "pure" control group receiving no intervention (or only usual care) and provides some control for the Hawthorne effect.

For both groups, frequency and content of each call is documented. Also, for both groups, intervention takes place in the first six months following baseline measures. This time frame was chosen given it takes that long for individuals with stroke to cope and adapt to their new reality [38] as well as to resume daily activities and social roles such as driving and leisure [16].

### Recruitment procedures and group allocation

**Figure 2:**
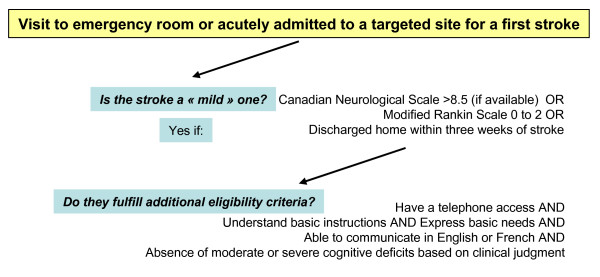
**Eligibility criteria**.

Daily, medical charts of all new stroke new admissions with a diagnosis of stroke and emergency room visits to any of the six targeted sites are reviewed by site-designated nurses to identify individuals with a potential first mild stroke. Once eligibility is ascertained, she explains the research to interested individuals and gives them a hard copy of the information and consent form thus giving time to potential participants to read more about the trial and think it over before providing consent. Informed consent is obtained upon the first phone contact by the research staff. If participants do have another stroke in the first month post-stroke, before baseline measures are collected and become ineligible, they are excluded. The site-designated nurse is also asked to document comorbidities based on information available in the medical chart. To ensure comparability between the two groups and using the information collected from the baseline measures, participants are being stratified according to the presence of important comorbidity (score ≥ 4 on the Comorbidity Index [37]) versus not (score < 4 on the Comorbidity Index [37]) and randomly allocated (ratio one-to-one) either to the WE CALL or YOU CALL group. A stratified block randomization procedure is done using a random-number generator on a computer and blocks of four. Randomization is carried out by a person who is not involved in the research [39]. Inclusion and exclusion criteria can be found in Figure 2.

### Primary and secondary outcome measures

The two primary outcomes are **unplanned-use of health services for negative events **and **quality of life**. Secondary outcomes include participation level, depressive symptoms and planned-use of health services for health promotion and secondary prevention. Unplanned use of health services for negative events is crucial to consider as a primary outcome as it reflects important health problems for individuals, is very costly to the individual and society and evidence suggests it can be decreased with adequate secondary prevention strategies. Quality of life is chosen as the other primary outcome, having emerged as *the ultimate outcome upon which the impact of stroke interventions and stroke programs should be evaluated *(Canadian Stroke Network). An economic evaluation comparing the two interventions will be also performed.

### Detailed description of outcome measures

1. **Unplanned-use of health services and planned-use **is being identified as the number of doctor visits, emergency or medical clinic visits, number of hospitalizations and any other use of other health care professionals (e.g. social worker, psychologist, physical therapist, occupational therapist, etc.) for the 6-month period of the study and an additional six months follow up period. To minimise recall bias, these data are being collected by way of a frequency calendar where each participant notes the health services they used, reasons (e.g. a fall, dizziness, stroke, follow up appointment, physical therapy, etc.) and most importantly if the visit was planned (scheduled) or not (emergency). At the time of data coding, each entry is being scrutinized by three stroke specialist health professionals, not involved in the study, to differentiate noise (such as emergency for a cold or planned-visits for foot care) versus any stroke-related use of the health care system (e.g. emergency for a fall, dizziness, or follow up visit for hypertension or any other risk factors) upon which the program aims to have an impact. Each entry is being dichotomised as either planned for health promotion/prevention or unplanned for a negative event. Using administrative data from each province was considered; however, given the high reliability between self-reported and administrative data of health-services use [40], we chose to rely on self-reported use only. Furthermore, this method of using a calendar has been successfully used with elderly individuals [40]. Given the high prevalence, especially in the early transition period following stroke onset, of stroke recurrence [41], medications errors [42,43]., of falls [44], depressive symptoms [45,46], etc., we estimate a mean number of unplanned-visit equal to three in the first six months post-stroke with a standard deviation of one.

2. A number of **quality of life **tools were reviewed (e.g. SF-36, Stroke Impact Scale, Quality of Life Index) and the tool chosen was a compromise between psychometric properties and adequacy of content for mild stroke. The 32 item questionnaire **Quality of Life Index **(QLI) [47] which was developed from Ferran's conceptual model of quality of life and which has been used with a stroke clientele [48] was chosen as the primary outcome. Each item of the QLI as relating to four life domains (health and functioning, socio-economic, psychological/spiritual and family), is evaluated in terms of satisfaction and importance on a six-point scale. Scores for each domain and a global score are expressed from 0-to-30, with a higher score indicating a better quality of life. These four life domains relate well with the main issues covered through the WE CALL intervention. Its has shown to have adequate psychometric properties (concurrent validity, test-retest reliability and high internal consistency: α = 0.90) [47] and thus should be responsive to therapy-induced change [49,50]. A one-point difference was observed in the first six months post-stroke descriptive follow-up (n = 63) for an effect size of 0.33 [51]. A two-point difference is considered a clinically meaningful change leading to a moderate effect size of 0.66. The **EQ-5D **which is composed of 5 items rated on a three-level scale [52] is also being completed as this questionnaire provides utility estimates needed to performed cost-utility analysis The EQ-5D is one of the recommended instruments by the Canadian Agency for Drugs and Technologies in Health (CADTH) [53].

3. The **LIFE-H **[54] is used to measure the **ICF participation domains **[55]. This questionnaire is composed of 77 items and covers 12 domains of participation [56]. The first six domains, Nutrition, Fitness, Personal care, Communication, Housing, and Mobility refer to the accomplishment of daily activities whereas the last six, Responsibilities, Relationships, Community life, Education, Employment and Recreation refer to the accomplishment of social roles. Participants are asked about the degree of difficulty in accomplishing the activity or the social role (without difficulty, with difficulty, by substitution or not realised) as well as assistance used (technical assistance, physical arrangements or human help). From their answers to these two simple questions, scores for each domain and a global score are derived and expressed from 0-to-9 where a higher score indicates better participation. It takes between 20-30 minutes to administer. The WE CALL intervention, through support and information about available community resources, is expected to foster improvements in many different domains of community reintegration covered by the LIFE-H, especially in regards to social roles. The LIFE-H has excellent psychometric properties [55,57], it has been extensively used in stroke [16,49,58-61] and is responsive to spontaneous recovery in individuals with "mild" stroke without showing ceiling effects [16]. An improvement of one-point out of a maximum score of nine is considered to be clinically important [55].

4. **Depression **is measured using the **Beck Depression Inventory II **(BDI-II) [62] created to correspond with the updated DSM-IV criteria for depression. It is composed of 21 items answered on a four-point Likert scale (score ranging 0 - 63) with a higher score indicating a greater severity of depression. A score of 0-13 is considered none or minimal range depression; 14-19 mild depression; 20-28 moderate depression; and 29-63 severe depression. Because of its relatively low reliance on somatic symptoms, the BDI is considered one of the more useful tools in assessing post-stroke depression [63]. It has strong psychometric properties: it has been shown to be responsive to change in 86 psychiatric patients treated with antidepressants [64] and was also as sensitive to change over time as DSM-IV criteria in a sample of 128 first-stroke followed for one year post-stroke [65]. A five-point difference is considered as a minimally important clinical difference [66].

Apart from the outcome measures indicated above, **sociodemographic data **(such as age, gender, living alone or not, socioeconomic status, etc.) are being collected at baseline. **Comorbidity **is also assessed at baseline by way of a medical chart review (by the site-designated nurse) using the Comorbidity Index [37] for stratification purposes. This index has been developed and validated for stroke outcomes research. It consists of a list of potential comorbidities rated on a four-point scale measuring how they impact on daily functioning: a score of four refers to major impact and it is therefore used as a threshold. All measures are available both in English and French.

### Sample size

Because the FITT study in the US was still underway at the time of writing this protocol, sufficient data on the unplanned-use of the health care system was not available. However, considering the high prevalence of stroke recurrence, emergency visits because of medications errors, falls and depression in the stroke population, we estimated a mean number of unplanned-use equal to three in the first six months post-stroke with a standard deviation of one unplanned-use. If we aim to decrease unplanned-use of the health care system by 10%, a sample size of 175 participants in each group is necessary with an alpha level set at 5% (for a bilateral test) and a power of 80%. A sample size of 57 individuals per group would be required to detect a two-point difference in the Quality of Life Index global score using data from a previous study [67] on quality of life of individuals with stroke (n = 72; mean = 20.91 ± 3.8). Given these estimates and considering a potential loss to follow up of 10%, a total sample of **384 individuals **is targeted for recruitment. These numbers are sufficiently large (using same parameters, required n < 65/group for participation and depression) to allow for large effect size of secondary outcomes and to do subgroup analysis according to comorbidity level.

### Planned statistical analyses

First, baseline characteristics of the sample will be presented using descriptive statistics. All analysis of primary and secondary available outcomes will be realised on an intention-to-treat basis thus respecting group allocation irrespective of whether participants received the intervention or not. The primary endpoint for analysis is the end of the intervention: six months period. A between group comparison on the difference in scores between six months measurement and baseline, between "we call" and "you call" will be made using an independent sample t-test for both primary and secondary outcomes. Effect size of the differences with their 95% confidence interval will be reported. As we anticipate unplanned-use of health services to not be normally distributed (we might end up with many 'zero' negative event), we plan to confirm parametric statistics with non-parametric testing. Also, repeated measures analysis will be realised using the four times of measurements (baseline, three months, six months and one year) where the between-subject factor is the intervention type, the within-group factor is time and their interaction provides the intervention effectiveness, including the one-year follow up phase. Also, multiple linear regression analyses will be used to explore the contribution of the potential explanatory variables (including group assignment, age, gender, living alone versus with a significant other, comorbidity level, *etc*.) on change in primary and secondary outcomes.

The economic impact of the two interventions "We call" and "You call" will be estimated with a cost-utility analysis. As one of the main outcomes of these interventions is their impact on quality of life, a cost-utility analysis represents the preferred type of economic evaluation. In a cost-utility analysis, outcomes are measured in terms of Quality Adjusted Life Years (QALYs) gained. In this study, QALYs will be estimated using the EQ-5D results measured at baseline, three months, six months and one year post-stroke. For this cost utility-analysis all costs associated with each intervention will be measured. These include the costs associated with the THCP for the "We call" group, costs associated with the THCP when contacted by a "You call" participant, cost of material provided and all costs of related health care resource utilisation (physician visits, emergency visits, use of other health care professionals, etc) for either negative event or secondary prevention. Cost associated with the use of the QLI, EQ-5D, LIFE-H, BDI-II and other procedures related to the study itself but not to the interventions will not be included. Result of this cost-utility analysis will be expressed in terms of incremental cost per QALY.

### Ethical considerations

This study received approval of Chatham Kent Health Alliance, Ontario (#08SE002) ethic review board on 2008-10-01; of Grand River Hospital, Kitchener-Waterloo, Ontario on 2009-01-12 (THREB #08-213); of Brant Community Health Care System, Brantford, Ontario in February 2009; of Calgary site in Alberta on January 2009 and of CRIR establishments for the province of Quebec on 2009-01-21 (CER # 373).

## Discussion

Improving our understanding of how to adequately support individuals with a first mild stroke will contribute to improved health service delivery to this oftentimes neglected group.

## Current study status

Enrolling participants in the six sites.

## Abbreviations

BDI: Beck Depression Inventory; EBM: Evidence based medicine; ICF: International Classification of functioning, disability and health; FITT: Family intervention telephone tracking; NIHSS: National Institutes of Health Stroke Scale; QUALYs: Quality adjusted life years; SPC: secondary stroke prevention clinic; SREBR: Stroke Rehabilitation Evidence-Based Review; THCP: trained health care professional; TIA: transient ischemic attack.

## Competing interests

The authors declare that they have no competing interests.

## Authors' contributions

AR is the lead author with close collaboration from NKB. All authors contributed to the development and writing of the study protocol. DB, RT, CW were more involved in the planning of the interventions. GB, JL, MK and SWD were more involved for methodological issues. RC, TG, SL, LHL were more involved for practical and feasibility issues. All authors read and approved the final manuscript.

## Pre-publication history

The pre-publication history for this paper can be accessed here:

http://www.biomedcentral.com/1471-2377/10/3/prepub
